# Cognitive flexibility in 12-month-old preterm and term infants is associated with neurobehavioural development in 18-month-olds

**DOI:** 10.1038/s41598-021-04194-8

**Published:** 2022-01-10

**Authors:** Yuta Shinya, Masahiko Kawai, Fusako Niwa, Yasuhiro Kanakogi, Masahiro Imafuku, Masako Myowa

**Affiliations:** 1 Graduate School of Education, The University of Tokyo, Tokyo, Japan; 2 Department of Pediatrics, Graduate School of Medicine, Kyoto University, Kyoto, Japan; 3 Graduate School of Human Sciences, Osaka University, Osaka, Japan; 4 Faculty of Education, Musashino University, Tokyo, Japan; 5 Graduate School of Education, Kyoto University, Kyoto, Japan

**Keywords:** Human behaviour, Cognitive control, Attention, Risk factors, Paediatric research

## Abstract

There is growing evidence that preterm children are at an increased risk of poor executive functioning, which underlies behavioural and attention problems. Previous studies have suggested that early cognitive flexibility is a possible predictor of later executive function; however, how it develops in infancy and relates to the later neurobehavioural outcomes is still unclear in the preterm population. Here, we conducted a longitudinal study to investigate oculomotor response shifting in 27 preterm and 25 term infants at 12 months and its relationship with general cognitive development and effortful control, which is a temperamental aspect closely associated with executive function, at 18 months. We found that moderate to late preterm and term infants significantly inhibited previously rewarded look responses, while very preterm infants did not show significant inhibition of perseverative looking at 12 months. Moreover, lower inhibition of perseverative looking was significantly associated with lower general cognitive development and attentional shifting at 18 months. These findings suggest that the early atypical patterns of oculomotor response shifting may be a behavioural marker for predicting a higher risk of negative neurobehavioural outcomes, including attention-related problems in preterm children.

## Introduction

The survival rate of very preterm (gestational age < 32 weeks) and/or very-low-birthweight (birth weight < 1,500 g) infants has increased due to the medical advancements in perinatology^1–3^; however, they have higher risks of behaviour and academic problems that persist into school age and young adulthood^4,5^. Furthermore, attention-related problems are prominent in these children^6–9^, which is also manifested in a two- to four-fold higher risk of attention deficit/hyperactivity disorder in this population^10,11^.

Several studies have shown that these problems in preterm populations arise partly from deficits in executive function [EF]^4,6,12,13^, which consists of cognitive skills to set goals and coordinate thoughts, emotions, and actions toward those goals^14,15^. Very preterm and/or very-low-birthweight children have been reported to show poor EF skills, including lower verbal frequency, working memory, and cognitive flexibility, from early childhood^16^ to young adulthood^6,17^. These studies suggest that deficits in EF have prolonged adverse effects on daily life achievements in preterms^18^. Therefore, it is crucial to identify early behavioural markers that are associated with the risk of executive dysfunction in people born prematurely.

From a developmental viewpoint, the emergence of EF has been observed in infancy and toddlerhood, while EF develops prominently throughout early childhood^15,19^. Much knowledge has been accumulated on the precursors and predictors of EFs (e.g. control of attention, self-regulation and reactivity, processing speed, and cognitive flexibility) during the first three years of life^14^. Cognitive flexibility involves the ability to update a task set with new information relevant to that task to achieve a goal and plan the next response accordingly or shift to a completely new task set. From preschoolers to adulthood, it has been demonstrated that cognitive flexibility can be dissociated into two separable latent variables: updating-specific (i.e. working memory) and shifting-specific^19,20^.

Shifting (switching) is an ability associated with cognitive flexibility and has traditionally been well studied using the A-not-B task in infancy^21,22^. In this task, a child is prompted to reach a certain location (A) to retrieve a hidden toy. After several attempts at A, the hidden location is switched to a new location (B). Thus, the child must shift their response from one rule to another rule by inhibiting the dominant response learned in the A trials, where the task is also thought to involve inhibitory control and working memory^22–24^. In neuroimaging studies, the performance of response shifting tasks has been shown to link the integrity and function of the prefrontal cortex, especially the dorsolateral prefrontal cortex (DLPFC)^25,26^. A recent study using path analysis has shown that A-not-B performance at 10 months is continuously associated with performance on EF tasks through age 6 and predicts verbal IQ and reading achievement at age 6^27^.

In preterm populations, the results of the few studies regarding cognitive flexibility, including shifting, are inconsistent during infancy^28–32^. It has been reported that preterm infants, especially very preterm infants, showed lower performance on the A-not-B task (i.e. more errors in perseveration) than term infants^28,29^. However, Matthew et al.^30^ reported that low-risk preterm infants outperformed term infants in reaching and looking versions of the A-not-B task at 6–14 months. Hodel et al.^31^ also reported that there was no significant group difference between moderately-to-late preterm and term infants, although lower gestational age was associated with lower performance on the A-not-B task.

This discrepancy in results may be explained by the differences in the severity of medical complications between the samples (e.g. very preterm birth and severe brain damage)^9,32^. However, it should be noted that the performance on the above behavioural face-to-face tasks may be susceptible to other developmental aspects known to be atypical among preterms, such as early poor motor skills^33^ and decreased sensitivity to social stimuli^34–36^. Considering that the experimenter’s social signals (e.g. eye contact^37^) or human actions^38^ elicit more perseverative errors in the A-not-B task, the lower sensitivity to such social signals in preterm infants may reduce the ability to detect the potential difference in response shifting from term infants when using the face-to-face task.

Therefore, a non-face-to-face eye-tracking study may be useful in controlling for the above confounding factors to reveal the development of response shifting, especially in preterm infants. Oculomotor response (i.e. eye movement) is a well-researched response that infants can control early in their development. In an oculomotor response shifting task using non-face-to-face eye-tracking (i.e. cognitive control task^39^), infants are first required to learn a predictable stimulus sequence (pre-switch phase), and in the next phase, they are required to inhibit their previously learned look response to learn a new conflicting look response (post-switch phase). Thus, this task has been used in recent studies as an early measure of emergent EF in a variety of contexts^31,39–42^. To date, only one study has used non-face-to-face eye-tracking to investigate early response shifting in preterm infants. In a study assessing early EF in moderately-to-late preterm infants^31^, their performance in the response shifting (reversal learning) task was comparable to that of term infants at 9 months. However, the sample did not include very preterm or extremely preterm infants who were at a higher risk for executive dysfunction. Moreover, it is still unclear how individual differences in shifting during the first year of life of preterm infants are related to higher risks of negative neurobehavioural outcomes, including deficits in EF.

This study investigated early cognitive flexibility related to emergent EF in preterm and term infants at 12 months of corrected age by the oculomotor response shifting task using a non-face-to-face eye-tracking technique. As most typical developing infants were reported to exhibit response shifting in previous studies using the A-not-B task until 12 months^22,24,27^, this period is assumed to be suitable for assessing individual differences in cognitive flexibility, including preterm infants. Furthermore, to reveal the significance of early cognitive flexibility in developing EF in preterm children, we longitudinally investigated the associations with parental reports of effortful control (EC) at 18 months of corrected age. EC is a temperamental aspect closely associated with individual differences in goal-directed control of attention and behaviour^43,44^. Although EC is primarily measured using parent reports, it has been indicated to have considerable overlap with behavioural measures for EF^45^. Additionally, given that the emergent EF, including response shifting, has been reported to relate to subsequent global cognitive functions^32^, we assessed the relationships to general cognitive development using a Japanese standardised developmental scale (i.e. Kyoto Scale of Psychological Development [KSPD];^46^).

Considering the previous studies indicating that preterm birth is associated with higher risks of deficits in EF^6,9^, it is possible that some preterm infants would exhibit a decreased ability to shift the oculomotor response (i.e. inhibiting perseverative looking at the previous target and shifting look to the new target) at 12 months. Furthermore, given that the ability to shift responses is an aspect of early cognitive flexibility related to developing EF, we predicted that the ability to inhibit and shift oculomotor response at 12 months would be positively associated with neurobehavioural outcomes, including effortful control at 18 months in samples comprising preterm infants.

## Method

### Participants

Thirty-four preterm infants (gestational age [GA] < 37 weeks) and 36 term infants (GA ≥ 37 weeks) participated in this study. All participants were recruited between 2013 and 2015 from the neonatal intensive care unit at Kyoto University Hospital, Japan, at term-equivalent age (i.e. postmenstrual age between 37 and < 42 weeks). They were partly included in previous studies conducted by our research group^34,35,47–49^. The inclusion criteria required that the subjects have no severe neurological complications, such as brain lesions (including periventricular leukomalacia, and grade III or IV intraventricular haemorrhage) and chromosomal abnormalities (see Table S1 for other demographic information on medical complications). All participants came from Japanese families and were considered middle class based on the census of their area of residence (Kyoto Prefecture): the Gini coefficient, which indicates disparity of income, was relatively small (0.28)^50^, and the average annual household income of the participants was around 5.3 million yen, which is almost the same as the average for the whole of Japan^51^.

After participating at term-equivalent age, 7 preterm and 11 term infants were excluded from this study because they could not participate in or complete the assessment at 12 months of corrected age due to hospital or family circumstances (e.g. no medical examination or moving; preterm: *n* = 6, term: *n* = 11) and infant circumstances (e.g. fussiness during assessment; preterm: *n* = 1). Therefore, the final sample consisted of 27 preterm and 25 term infants. Considering the large effects of gestational age on neurobehavioural development including EF^6,9,10,16^ and the inconsistencies in outcomes between very preterm (VP;^4–8,10,12^) and moderate-to-late preterm (MLP;^31,52–56^) groups, preterm infants were assigned to two subgroups according to gestational age: very preterm (VP) infants (GA < 32 weeks; *n* = 12) and moderate-to-late preterm (MLP) infants (GA ≥ 32 weeks but < 37 weeks; *n* = 15). The study was conducted with the approval of the ethics committee of Kyoto University Graduate School and Faculty of Medicine (No. E581), and according to the standards specified in the Declaration of Helsinki from 1964. Written informed consent was obtained from the participants’ parents when they were at term-equivalent age. The demographic data of the participants at term-equivalent age are shown in Table 1.

**Table 1:** Demographic information in preterm and term infants at birth at 12-month-old.

	Preterm infants						Term infants			*F*-value	*p*-value	Post hoc (*p* < .05)
	VP (*n* = 12)			MLP (*n* = 15)			Term (*n* = 25)					
	*M*	*SD*	*Range*	*M*	*SD*	*Range*	*M*	*SD*	*Range*			
Gestational age (weeks)	28.96	2.20	25.57–31.86	34.67	0.88	32.86–36	39.29	1.22	37.14–41.43	217.78	< .001	VP < MLP < FT
Birth weight (g)	1060	261	618–1640	1978	277	1381–2572	2956	366	2362–4110	148.61	< .001	VP < MLP < FT
Postnatal age (months)	14.83	0.42	14.36–15.51	13.45	0.63	12.68–15.15	12.79	0.55	11.04–14.39	56.48	< .001	VP > MLP > FT
Corrected age (months)	12.30	0.20	12.02–12.55	12.22	0.54	11.5–13.7	12.62	0.44	11.17–13.73	4.82	0.012	VP < FT, MLP < FT
SGA	2/12			3/15			1/25			1.37	0.264	
Female	7/12			3/15			12/25			3.50	0.038	VP > MLP, MLP < FT

VP = very preterm; MLP = moderate-to-late preterm; SGA = small-for-gestational age.

### Apparatus and stimuli

For eye-tracking measurement (i.e. oculomotor response shifting task) at 12 months, we used a Tobii TX300 (Tobii Technology, Stockholm, Sweden) near-infrared gaze tracking system to measure eye movement at 300 Hz and present movie stimuli to infants through a 23-inch display. Stimulus presentations and measurements were controlled using a laptop computer (Dell Precision M6600) with Tobii Studio software ver. 3.2.1 (Tobii Technology, Stockholm, Sweden). We set the minimum fixation duration in the Tobii I-VT fixation filter to 60 ms, as the duration resulted in a significant improvement in the accuracy of the fixations produced. All movies were edited using Adobe Premiere Pro CS5.5 (Adobe Systems Inc., San Jose, CA).

The oculomotor response shifting task was modelled in previous studies^39,42^. The infants were presented with a short movie clip for a total of 18 trials (nine each of pre-switch and post-switch), interleaved with short attention-getter stimuli. After the infants fixated on the attention-getter stimuli, the trial commenced following a 300 ms delay. Two white blank squares (10.10° × 10.10°) were presented left and right simultaneously with an auditory stimulus (either of the two electronic sounds) (the anticipatory period; duration, 2000 ms). A visual-audio reward (either of the two cartoon animals, 8.05° × 8.24°; duration, 2000 ms) then appeared on one side of the screen (in either the left or right). The reward was displayed on the same side of the screen for nine trials in a row during the pre-switch phase and on the other side for nine trials in a row during the post-switch phase. The auditory and reward stimuli were also switched to the other stimulus after the pre-switch phase. The presentation orders of the reward’s positions and stimulus and the auditory stimulus were counterbalanced across participants.

### Procedure

#### Eye-tracking for oculomotor response shifting task at 12 months

The cognitive task with preterm infants was conducted at Kyoto University Hospital, and that with term infants was conducted at Kyoto University. In each of those environments, visual distractions were removed as much as possible, and the ambient noise level in the room was judged to be perceptually ‘low’. Therefore, the testing conditions were appropriate for the eye-tracking test at each location^34,35,47^. Participants were seated on their parent’s lap with their eyes approximately 60 cm from the monitor, and were monitored through visual observation to check their constant attention to the monitor and fixation on the attention-getter stimuli by the web camera. Prior to recording, we adjusted the position of participants and eye-tracking monitor to evaluate their stable eye movement and performed a five-point calibration procedure before data collection which met the standard of the Tobii Studio software. Any unsuccessfully calibrated points were recalibrated. The oculomotor response-shifting task started immediately after a successful calibration procedure.

#### Assessment of general cognitive development and effortful control at 18 months

The infants participated in a follow-up study to assess general cognitive development and effortful control at 18 months of corrected age. General cognitive outcomes were evaluated using the KSPD^46^. The KSPD is a Japanese standardised developmental scale commonly administered to typically developing infants and low-functioning children with disabilities, including preterms^57–59^. The KSPD measures general developmental progress and delays in the following three domains: postural-motor (P-M), cognitive-adaptive (C-A), and language-social (L-S). The developmental quotients are highly correlated with the corresponding composite scores (i.e. motor, cognitive, and language) on Bayley III^59^. The KSPD assessment was conducted in an examination room at Kyoto University Hospital for preterm infants and in an experimental room at Kyoto University for term infants. These data were also used to relate to neonatal behavioural characteristics in our previous study^49^. As a temperamental aspect of emergent EF^43–45^, effortful control was evaluated using the Japanese version of the Early Childhood Behavior Questionnaire (ECBQ)^60^. In ECBQ, effortful control (EC) is a validated temperament factor defined primarily by loadings of inhibitory control (IC), attention shifting (AS), low-intensity pleasure (LIP), cuddliness (Cu), and attention focusing (AF)^44^. After the KSPD assessment, the parents were given a questionnaire sheet of the ECBQ and were asked to complete and return it by post. These instruments were used to assess the frequency over the past one or two weeks of temperament related behaviours on a 7-point Likert scale (1 = never; 7 = always).

For the follow-up assessment at 18 months from the initial sample of 52 infants, 47 infants completed the KSPD (VP group, *n* = 12; MLP group, *n* = 14; and term group, *n* = 21) and 40 infants were assessed by the ECBQ (VP group, *n* = 9; MLP group, *n* = 11; and term group, *n* = 20). At the KSPD assessment, the mean corrected age of the VP group was 18.33 months (*SD* = 0.32, range = 17.71 − 19.06), 18.34 months for the MLP group (*SD* = 0.81, range = 16.76 − 19.68), and 18.25 months for the term group (*SD* = 0.54, range = 17.15 − 19.78). At the ECBQ assessment, the mean corrected age of the VP group was 18.69 months (*SD* = 0.62, range = 18.17 − 19.81), 18.92 months for the MLP group (*SD* = 0.78, range = 18.37 − 20.47), and 18.48 months for the term group (*SD* = 0.38, range = 17.68 − 19.12). A few participants did not complete the follow-up assessments for the following reasons: (KSPD: refusal to participate [e.g. moving far away; term: *n* = 3], fussiness or crying during the assessment [preterm: *n* = 1, term: *n* = 1]; ECBQ: refusal to participate [term: *n* = 3], forgetting to turn in or incomplete responses to questionnaires [preterm: *n* = 8, term: *n* = 1]).

### Data analysis

#### Oculomotor response shifting task at 12 months

Eye movement data were analysed using the Tobii's standard statistics package. Areas of interest (AOIs) were defined to determine each infant’s duration of anticipatory looking. The AOIs consisted of left and right areas, which covered the two white blank squares (10.10° × 10.10°).

We calculated the infants’ looking time towards the whole display and the AOIs during the 2-s time window around the anticipatory period. The time window was defined as starting 300 ms after the appearance of the two white blank squares and ending 300 ms after the appearance of the reward, based on previous studies^42^. To investigate the reduction in looking at the previous target and increase in looking at the new target during the post-switch phase, we measured looking time at correct/incorrect target AOIs as dependent variables. Based on a previous study of the looking versions of the A-not-B task^61^, both looking time measures during the post-switch phase are assumed to relate to response shifting, a precursor of executive functioning. Specifically, increased correct looking time would be related to shifting looks to the new target, while decreased incorrect looking time would be related to inhibiting perseverative looks. While some previous studies employed discrete categorical variables such as collect anticipatory looks^39^ (see Supplement S1 for the results of categorical discrete variables of anticipatory looks), these variables are assumed to collapse potentially valuable individual differences in cognitive processes^62^. Therefore, we focused on the looking time measures as representative values associated with individual differences in response shifting and developmental outcomes.

Furthermore, for the comparisons to specifically investigate the temporal changes of oculomotor responses, the trials, except for the first, were grouped into two blocks (i.e. first [2–5 trials]/second [6–9 trials]), similar to previous research^39^. Although Kovács & Mehler^39^ divided each phase into three blocks to examine group differences in response shifting, due to the low statistical power we employed two block divisions. The first trial was excluded because infants could not predictably look at the reward position during the anticipatory period of the first trials at each phase. The looking time was averaged for each block in the pre- and post-switch phases. Within the pre- or post-switch phase, infants with more than three trials in which they did not look at the display during the anticipatory period were excluded from the analyses (*n* = 1).

Furthermore, we analysed each looking time measure with a 3 (group: VP, MLP, Term) × 2 (block; first, second) factorial analysis of variance (ANOVA). The factor of group was between-subjects, and the factor of block was within-subjects. In repeated-measures ANOVAs, post hoc multiple comparisons were conducted using Shaffer’s modified sequentially rejective Bonferroni procedure. A sensitivity power analysis was also conducted in G*Power^63^ to assess the detectable range of effect sizes based on the given sample size (*n* = 52), power (0.80), and alpha (*p* = 0.05). The sensitivity power analysis revealed that the minimum effect sizes that could be detected with the sample size were *η*^*2*^ = 0.136 (group effect), 0.030 (block effect), 0.039 (interaction effect), 0.165 (simple main effect of group), and 0.136, 0.108, and 0.064 (simple main effect of block, respectively, in VP, MLP, Term) (for the reference values^64^ of *η*^*2*^: small (0.01), medium (0.06), and large (0.14) effect sizes).

#### General cognitive development and effortful control at 18 months

For general cognitive functioning at 18 months, the developmental quotient was calculated by dividing the developmental age as measured by the KSPD by corrected age and multiplying the resulting quotient by 100 for each of the three areas (P-M, C-A, and L-S). For ECBQ assessment at 18 months, in addition to the composite score of EC, we used the five sub-factors related to IC, AS, LIP, Cu, and AF from the ECBQ. For reference, the mean scores of ECBQ in typically developing Japanese populations at 18–24 months [60, *n* = 128] were shown as follows: EC, *M* (*SD*) = 4.21 (1.01); IC, *M* (*SD*) = 3.29 (0.98); AS, *M* (*SD*) = 4.56 (0.67); LIP, *M* (*SD*) = 4.64 (0.76); Cu, *M* (*SD*) = 4.74 (0.74); AF, *M* (*SD*) = 3.82 (1.01). For each developmental score, we performed a one-way ANOVA (group; VP, MLP, Term) with post hoc multiple comparisons using Shaffer’s modified sequentially rejective Bonferroni procedure. A sensitivity power analysis based on the given sample size (KSPD: *n* = 47, ECBQ: *n* = 40) showed that the minimum effect size detected with the sample sizes was *η*^*2*^ = 0.180, and 0.207, respectively.

#### Relations between inhibition of perseverative looking at 12 months and neurobehavioural development at 18 months

We performed partial correlation analyses to investigate the independent contributions of the performance (i.e. looking time on consistent/inconsistent AOIs during the post-switch phase) on the oculomotor response shifting task at 12 months to cognitive development and effortful control at 18 months. We calculated the partial correlation coefficients controlling for gestational age^6^, sex (female = 0, male = 1), corrected age at 12 months (assessment of response shifting task), and 18 months (assessment of KSPD/ECBQ) as confounding factors affecting developmental outcome variables. In this analysis, we excluded other demographic variables related to gestational age (i.e. birth weight and postnatal age) to avoid collinearity of predictors. In addition, as in previous studies in developmental psychology or paediatrics^35,65–67^, we prioritised describing as many potential developmental relationships as possible. Therefore, in the analysis, we did not apply multiple testing corrections, which lead to a substantial reduction in statistical power^68,69^. A sensitivity power analysis based on the given sample sizes *n* = 47 for KSPD and *n* = 40 for ECBQ showed that the minimum effect detected on the sample sizes was *r* = 0.385 and 0.414, respectively (for the reference values^64^ of *r*: small (0.10), medium (0.30), and large (0.50) effect sizes).

## Results

### Comparisons of looking time in oculomotor response shifting task at 12 months

As a preliminary analysis, we checked the effects of group and block on looking times toward the whole display during the anticipatory phase. For the pre-switch phase, we did not find group and interaction effects, while the block effect was significant (first > second; *F*_*1,49*_ = 9.07, *p* = 0.004, *η*^*2*^ = 0.030). For the post-switch phase, there were no significant group, block, or interaction effects. These results indicated that their attention to the task during the anticipatory period did not differ among groups regarding gestational age.

**Figure 1:**
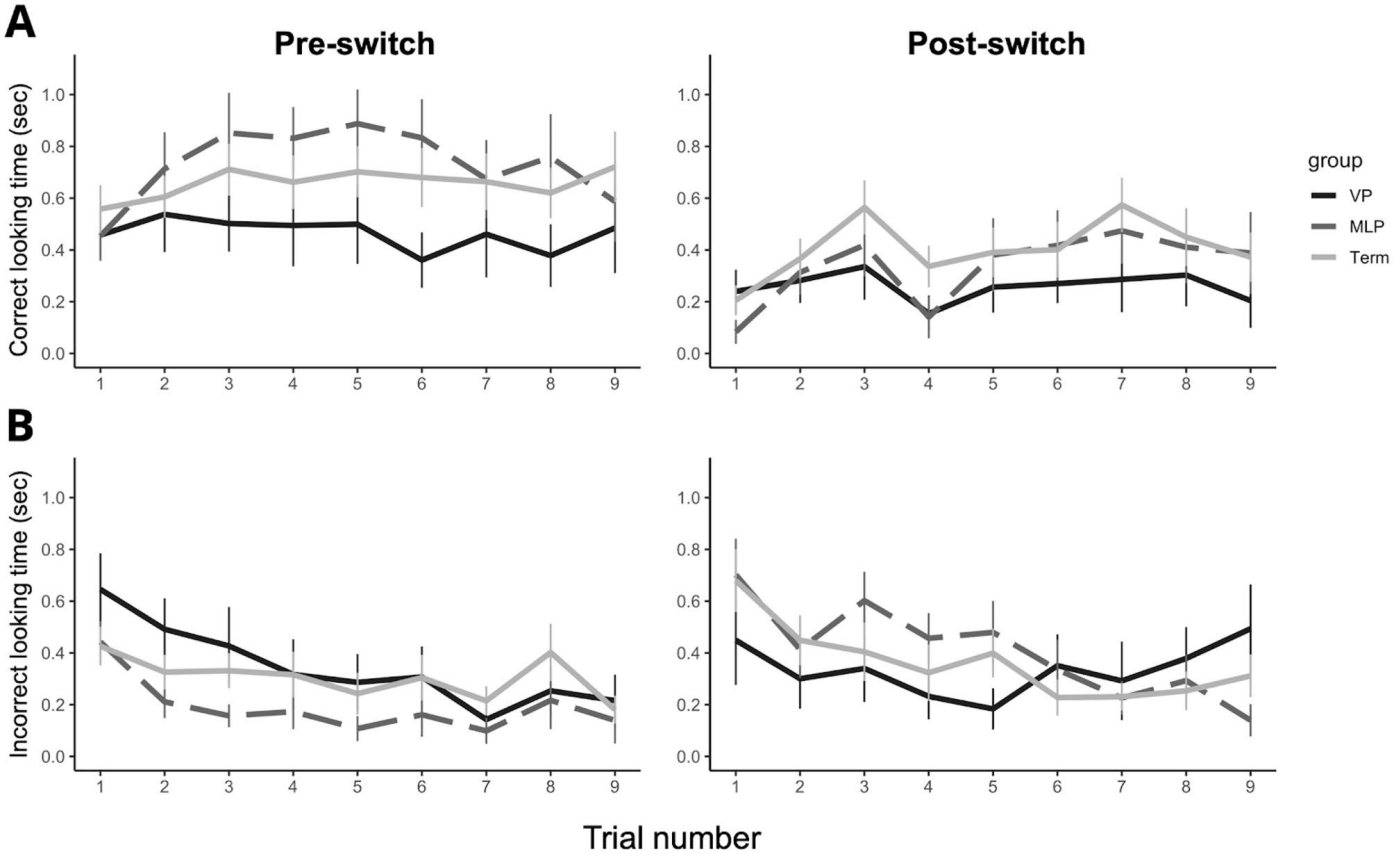
Change of looking time on (**A**) correct target and (**B**) incorrect target in VP (*n* = 12), MLP (*n* = 15), and Term infants (*n* = 25) across 18 trials (Pre-switch phase: 1–9 trials; Post-switch phase 1–9 trials). Error bars indicate 1 standard error of the mean.

**Figure 2:**
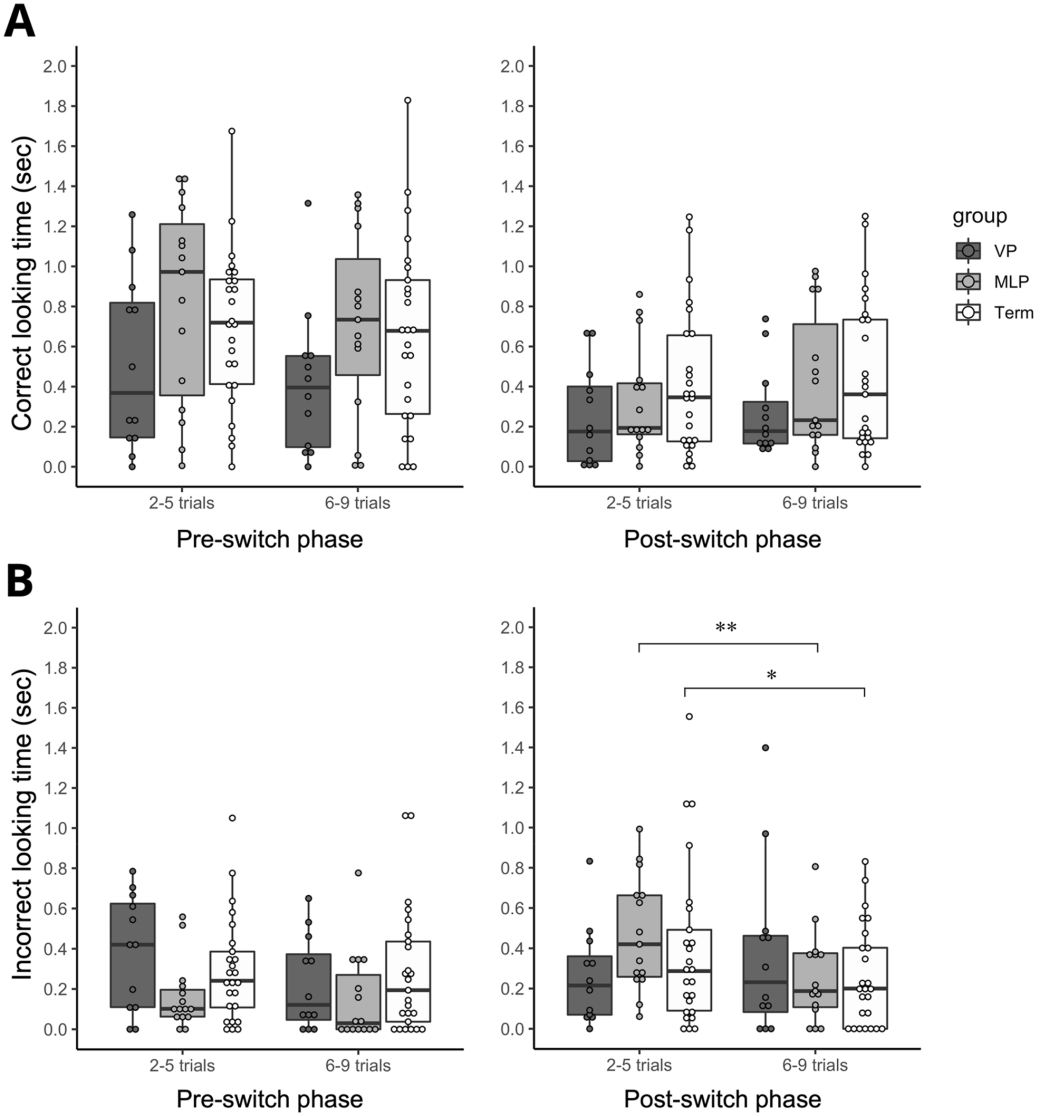
Box and scatter plots indicating comparisons of looking time on (**A**) correct target and (**B**) incorrect target between the former (2–5 trials) and latter half (6–9 trials) of the pre/post-switch phase in VP (*n* = 12), MLP (*n* = 15), and term infants (*n* = 25). Error bars indicate 1 standard error of the mean. **p < .001, *p < .05.

The temporal changes in looking time on correct/incorrect target AOIs for each gestational age group (VP, MLP, and Term) across all trials are shown in Fig. 1. Comparisons of mean looking time on correct/incorrect target AOIs across the first and second blocks of each phase and the group are shown in Fig. 2. For the correct target AOI, there were no significant group, block, or interaction on looking time at any of the pre -and post-switch phases (all *ps* > 0.10; Fig. 2A). However, for the incorrect target AOI during the post-switch phase, we found a significant block (*F*_*1,49*_ = 6.86, *p* = 0.012, *η*^*2*^ = 0.026) and interaction effects on looking time (*F*_*2,49*_ = 4.90, *p* = 0.012, *η*^*2*^ = 0.037). Simple main effect analyses revealed that MLP and Term infants showed significant decreases in looking time toward the incorrect AOI (MLP: *F*_*1,14*_ = 21.96, *p* < 0.001, *η*^*2*^ = 0.164; Term: *F*_*1,24*_ = 4.50, *p* = 0.044, *η*^*2*^ = 0.040), although there was no significant change in the looking time target in the VP group (*p* = 0.225, *η*^*2*^ = 0.027) (Fig. 2B). These results indicate that MLP and Term infants, except VP infants, could significantly inhibit perseverative looking towards the incorrect target across the trials of the post-switch phase.

### Differences in neurobehavioural development at 18 months among Preterm and Term infants

The developmental scores of the KSPD and the composite and subscale scores of the ECBQ at 18 months of corrected age are shown in Table 2. For the KSPD scores, there were significant group differences in the L-S score (*F*_*2,44*_ = 6.78, *p* = 0.003, *η*^*2*^ = 0.236) but marginal differences in the C-A score (*F*_*2,44*_ = 3.11, *p* = 0.055, *η*^*2*^ = 0.124). Post hoc testing showed that the VP group had a lower L-S score than the MLP group (*p* = 0.002) and the Term group (*p* = 0.009). Furthermore, the C-A score of the VP group was also marginally lower than that of the MLP and Term groups (both *p* = 0.061). For the ECBQ scores, there was a significant group difference only in the IC score (*F*_*2,37*_ = 3.26, *p* = 0.049, *η*^*2*^ = 0.150). The VP group showed significantly lower IC than the MLP group (*p* = 0.046) and marginally lower IC than the term group (*p* = 0.098).

**Table 2:** Developmental outcomes in preterm and term infants at 18-month-old.

	Preterm infants						Term infants			*F*-value	*p*-value	Post hoc (*p* < .05)
	VP			MLP			Term					
	*M*	*SD*	*Range*	*M*	*SD*	*Range*	*M*	*SD*	*Range*			
*KSPD*	(*n* = 12)			(*n* = 14)			(*n* = 21)					
Postual-Moter	105	16	87–151	107	13	91–135	102	15	63–129	0.58	0.565	
Cognitive-Adaptation	101	15	70–121	111	7	99–127	109	10	81–122	3.11	0.055	
Language-Social	92	12	59–116	109	10	93–124	103	11	75–120	6.78	0.003	VP < MLP, VP < Term
*ECBQ*	(*n* = 10)			(*n* = 9)			(*n* = 21)					
Effortful Control	4.07	0.53	3.27–4.69	4.53	0.54	3.96–5.52	4.21	0.53	2.84–5.07	1.86	0.170	
Inhibitory Control	2.64	0.98	1.00–4.17	3.85	0.98	2.50–5.44	3.15	1.08	1.00–5.00	3.26	0.049	VP < MLP
Attention Shifting	4.34	0.49	3.75–5.09	4.89	0.74	4.10–6.25	4.72	0.72	3.64–6.73	1.73	0.191	
Low-intensity Pleasure	4.41	0.58	3.45–5.18	4.78	0.69	3.80–5.82	4.65	0.58	3.45–5.80	0.95	0.396	
Cuddliness	4.97	0.73	4.08–6.08	4.84	0.30	4.42–5.25	4.71	0.89	2.25–6.00	0.41	0.664	
Attention Focusing	4.08	1.46	1.75–7.00	4.37	1.29	3.12–7.00	3.76	0.80	2.67–5.50	1.01	0.373	

VP = very preterm; MLP = moderate-to-late preterm; KSPD = Kyoto Scale of Psychological Development; ECBQ = The Early Childhood Behavior Questionnaire.

### Relationship of oculomotor response shifting at 12 months to neurobehavioural development at 18 months

Furthermore, we investigated how oculomotor response shifting during the post-switch phase at 12 months would be associated with neurobehavioural development, including general cognitive development and effortful control at 18 months. First, correlation analysis indicated that the looking time on an incorrect target AOI during the 1st block (2–5 trials) was positively associated with the IC in the ECBQ (*r* = 0.57, *p* < 0.001). We also found that the looking time during the 2nd block (6–9 trials) was negatively related to the C-A and L-S in the KSPD (*r* = -0.35, *p* = 0.016; *r* = -0.38, *p* = 0.008) and the AS in the ECBQ (*r* = -0.31, *p* = 0.049). Furthermore, partial correlation analysis revealed that these correlations remained significant after controlling for gestational age, sex, and corrected age at assessment (Table 3). These results indicated that the occurrence of perseverative looking at 12 months was significantly correlated with higher inhibitory control at 18 months. Moreover, the inhibition of perseverative looking at 12 months predicted higher cognitive and social developmental outcomes and attention shifting at 18 months. However, the looking time on the correct target AOI was not significantly associated with any developmental outcome at 18 months (all *ps* > 0.05).

**Table 3:** Correlations and partial correlations of looking time (LT) during Post-switch phase on oculomotor response shifting task to neurobehavioural development at 18-month-old.

	KSPD (*n* = 47)			ECBQ (*n* = 40)					
	P-M	C-A	L-S	EC	IC	AS	LIP	Cu	AF
Correct LT (post 2–5 trials)	−.22 (−.18)	.06 (−.01)	.27 (.21)	−.01 (.00)	−.06 (−.07)	−.02 (−.03)	.28 (.23)	−.12 (−.06)	−.06 (−.04)
Correct LT (post 6–9 trials)	−.22 (−.18)	−.03 (−.08)	.08 (.05)	.01 (.02)	.02 (.03)	.04 (.05)	.17 (.12)	−.17 (−.14)	.04 (.05)
Inccorect LT (post 2–5 trials)	.00 (.03)	−.15 (−.20)	−.17 (−.28)	.26 (.13)	.57***(.49**)	.07 (.02)	.14 (.07)	−.06 (.04)	−.03 (−.08)
Inccorect LT (post 6–9 trials)	−.07 (−.13)	−.35* (−.34*)	−.38* (−.36*)	−.05 (−.15)	.30 (.20)	−.31* (.36*)	−.08 (−.12)	.00 (−.08)	−.14 (−.18)

## Discussion

This study longitudinally investigated oculomotor response shifting as an early measure of cognitive flexibility in preterm and term infants at 12 months of corrected age and its relationship to neurobehavioural development at 18 months of corrected age. We found that MLP and term infants significantly inhibited previously rewarded look responses, while we found that the VP infants showed decreased inhibition of perseverative looking at 12 months. As expected, these findings partly support our hypothesis that some preterm infants would exhibit a decreased ability to shift oculomotor response at this age. Furthermore, consistent with our prediction, the lower inhibition of perseverative looking was later associated with negative outcomes in general cognitive development and attentional shifting at 18 months. Thus, these findings provide new evidence suggesting that the atypical oculomotor response shifting in infancy may be associated with higher risks of later negative neurobehavioural outcomes, including deficits in EF, in preterm children.

For the oculomotor response shifting task at 12 months, MLP and term infants at 12 months exhibited looking to the previously rewarded target (i.e. perseverative looking) during the first block (2–5 trials) of the post-switch phase, and significantly decreased the perseverative looking during the second block (6–9 trials). However, VP infants did not show a significant decrease across the first to second blocks during the post-switch phase. Since there were no differences between the three gestational groups in attention to the display during the anticipatory period, the differences in the inhibition of perseverative looking may not simply be due to attention to the task or arousal state. Our results seem to correspond with some previous studies reporting lower response shifting in the A-not-B task in preterm infants^28,29^. In a previous study using a similar task to ours (i.e. cognitive control task;^39–42^), the quick inhibition of perseverative looking after switching a reward’s position is assumed to be a precursor of emergent EF during the first year of life. Given this assumption, it is possible that the lack of the inhibition in the VP infants in our sample may reflect the precursor of later executive dysfunction^4,6,9,16^, which involves deficits in higher cortical areas, such as the DLPFC^25,26^.

This possibility may also be supported by our developmental relationship results. We found that longer perseverative looking during the second block of the post-switch phase was significantly associated with developmental delays in both cognitive and social domains of KSPD and lower attentional shifting, which is a sub-factor of effortful control assessed by parental report of temperament scale (ECBQ), at 18 months of corrected age. These findings correspond with previous studies reporting that the performance of the A-not-B task in infancy predicted later general cognitive function^32^ and EF^27^. Therefore, it is possible that the early cognitive flexibility observed in this study may be associated with later executive dysfunction^6,12,13,16^ and attention-related problems^4,6,7,9–11^ in preterm populations.

However, the atypical pattern of perseverative appearance observed in the VP infants may be partly due to decreased learning of the correct target’s position during the pre-switch phase, given previous studies reporting slower target fixation and lower processing speed in preterm infants^70,71^. VP infants tended to spend less time looking at the correct target in the pre-switch phase and the incorrect target in the first block of the post-switch phase, although these tendencies were not significant (see Table S6 for correlations among the looking time measures). This interpretation is consistent with theories that explain infantile perseverative behaviours as the result of a system that builds stability by bringing past activities into the present (parallel distributed processing model^23^; dynamic field theory^24,72^). In previous research on the A-not-B error task, the perseverative errors decreased from 7 or 8 to 12 months^22^; nevertheless, at 5–6 months infants have been reported to produce errors less frequently than at 8 months^72^, suggesting that there is a developmental trend from non-perseveration to perseveration early in the acquisition of a skilled behaviour. The authors speculate that the reason could be that younger individuals are not affected by their own recent activity and their response is tightly tied to the current moment, due to a lack of stable motor or perceptual memory.

Given the above theoretical explanations, in the present study, perseverative looking in the first half of the post-switch phase may represent the stable memory of the reward position in the pre-switch phase, while that in the second half may represent a lack or decrease of inhibition, leading to the differences in developmental associations with neurobehavioural outcomes. We found that incorrect looking in the first half of the post-switch phase, unlike in the second half, was positively correlated with the inhibitory control of ECBQ at 18 months of corrected age. Inhibitory control refers to the ability to ignore distractions and focus on the most relevant aspects of the environment (e.g. reward), even in infancy^15,73^. Therefore, it is possible that infants with higher inhibitory control would exhibit more perseverative looking immediately after switching targets, due to the strong memory by higher focusing on the previously rewarded target during the pre-switch phase. Considering these findings, it may be necessary to measure both the occurrence and inhibition of perseverative behaviours in response shifting tasks to predict later neurobehavioural outcomes, including effortful control.

In contrast to the VP group, the MLP group did not differ from the term group in the inhibition of perseverative looking; rather, the pattern was more pronounced than the term infants. A similar trend was also observed in neurobehavioural outcomes (i.e. KSPD and ECBQ) at 18 months. Although some previous studies reported that children born MLP appear to have equivalent cognitive function, including EF, compared to term children^52–54^, recent studies have reported that MLP children have slightly higher risks of poor school performance^55,56^ and EFs^31^. For example, Hodel et al.^31^ reported that there were no significant differences in some emergent EF skills, including performance on the A-not-B task and the oculomotor response shifting task, between MLP and term infants at 9 months of corrected age, consistent with our results; however, they also revealed that poorer performance on most of the emergent EF skills was related to lower gestational age. However, little is known about how their behavioural and cognitive characteristics from infancy would relate to the risk of later executive dysfunction in the MLP group; thus, long-term follow-up is needed to reveal this issue in the future.

It should also be noted that the incorrect looking time during the post-switch phase was more sensitive to shifting oculomotor responses at 12 months, compared to the correct looking time or the anticipatory looks^39^ (see also Supplement S1). Conceptually, both increased correct looking and decreased incorrect looking are assumed to relate to the ability to shift oculomotor response, a rudimentary form of EFs^61^. Nevertheless, the incorrect looking was not necessarily the perfect counterpart to the correct looking (see Table S6), and may more directly represent the degree of perseverative looking and its inhibition than the other measures in the task. In addition, these continuous measures may have more information about individual task performance related to response shifting than discrete categorical measures, such as the anticipatory looks^62^. Given these points, in the oculomotor response shifting task, it is important to examine in more detail the similarities and differences in cognitive processes reflected by these looking measures.

Our findings provide several potential implications for clinical applications in preterm children from infancy to toddlers. Given the developmental relationships revealed in the current study, early assessment of perseverative looking in oculomotor response shifting may be useful as an additional marker to detect the high risks of executive dysfunction in preterm children. In addition, some recent studies have shown that early training of attentional control in infancy can have short-term effects on emergent EF^42,74,75^. Therefore, it is possible that interventions including oculomotor response shifting tasks, especially those which involve inhibiting perseverative looking, have positive effects on the subsequent neurobehavioural development in preterm children^76^. Given the potential cascade of early negative effects on neurobehavioural outcomes in high-risk preterms^71,77^, it is important to consider whether such intervention reduces the risk of long-term executive dysfunction.

One of the limitations of this study is the relatively small sample size; each group of preterm infants was smaller than that of term infants. According to the sensitivity analyses, some of the minimum effect sizes that could be detected with the sample size were relatively large^64^. Therefore, considering that some analyses were powered to detect large effect sizes but not moderate or small effects, our null results should be viewed with caution as there is a possibility of Type II error. In addition, the atypical pattern of perseverative looking observed in the VP group might be due to various profiles of preterm infants (see Supplement S2 for the relationship of medical complications during the neonatal period). Given the low statistical power and potential confounding factors, the results should be confirmed in a larger sample in the future. Second, the developmental outcomes of effortful control at 18 months were assessed using parental reports. Direct independent observation in the controlled laboratory would be desirable to exclude the effects of subjective parental bias, although parental reports have some benefits, such as capturing children’s behaviours related to parental difficulties in daily life^43–45^. Third, we only assessed developmental outcomes up to a modified 18 months. Considering that the development of EF is remarkable across childhood^15,19^, it is necessary to investigate the longer-term relationship of perseverative looking in infancy and EF in early childhood and school age.

In conclusion, this study revealed the atypical characteristics of early cognitive flexibility in preterm infants at 12 months of corrected age using the oculomotor response shifting task based on non-face-to-face eye-tracking. In particular, very preterm infants exhibited a lack of inhibition of perseverative looking to the former rewarded target. Furthermore, we found that lower inhibition of perseverative looking was associated with lower general cognitive development and attentional shifting at 18 months. These findings suggest that the early assessment of perseverative looking in oculomotor response shifting would be a behavioural marker associated with higher risks of later negative neurobehavioural outcomes, including attention-related problems in preterm populations.

## Supplementary Information

Supplementary Information.

## Data Availability

All data analysed during this study are available from the corresponding author upon reasonable request.
