# Supplementary material for: Cognitive flexibility in 12-month-old preterm and term infants is associated with neurobehavioural development in 18-month-olds
Source: Sci Rep. 2022 Jan 10;12:3. doi: 10.1038/s41598-021-04194-8 (PMC8748813; doi:10.1038/s41598-021-04194-8)
Supplement: Supplementary file 1 — Supplementary Information. [file 41598_2021_4194_MOESM1_ESM.docx]

**Cognitive flexibility in 12-month-old preterm and term infants is associated with neurobehavioural development in 18-month-olds**

Yuta Shinya^*^, Masahiko Kawai, Fusako Niwa, Yasuhiro Kanakogi, Masahiro Imafuku, & Masako Myowa

* Correspondence concerning this article should be addressed to Yuta Shinya

E-mail: shinya@p.u-tokyo.ac.jp

This file includes:

- Supplement S1: Categorical data for anticipatory looks in the oculomotor response shifting task for 12 months

- Supplement S2: Relationships of birth profile to oculomotor response shifting at 12 months and neurobehavioural outcomes at 18 months

- Demographic data on medical complications (Table S1)

- ANOVA tables for looking time on oculomotor response shifting task at 12 months (Table S2-5)

- Correlations among looking time measures on oculomotor response shifting task at 12 months (Table S6)

**Supplement S1: Categorical data for anticipatory looks in the oculomotor response shifting task for 12 months**

In addition to the continuous variables of looking time which we reported in the main text, we assessed the categorical discrete variables for anticipatory looks reported in previous research (e.g. [1]).

We coded when infants looked at the correct or incorrect areas of interest (AOI) during the 2 sec time window starting 300 ms after the appearance of the two white blank squares and ending 300 ms after the appearance of the reward for each trial [2]. Trials where the infant showed a longer looking time at the correct AOI were defined as correct anticipatory looks, whereas those where the infant showed longer looking to the incorrect AOI were defined as incorrect anticipatory looks. Changes in the proportion of correct anticipatory looks across trials in pre- and post-switch phases are shown in **Figure S1**.


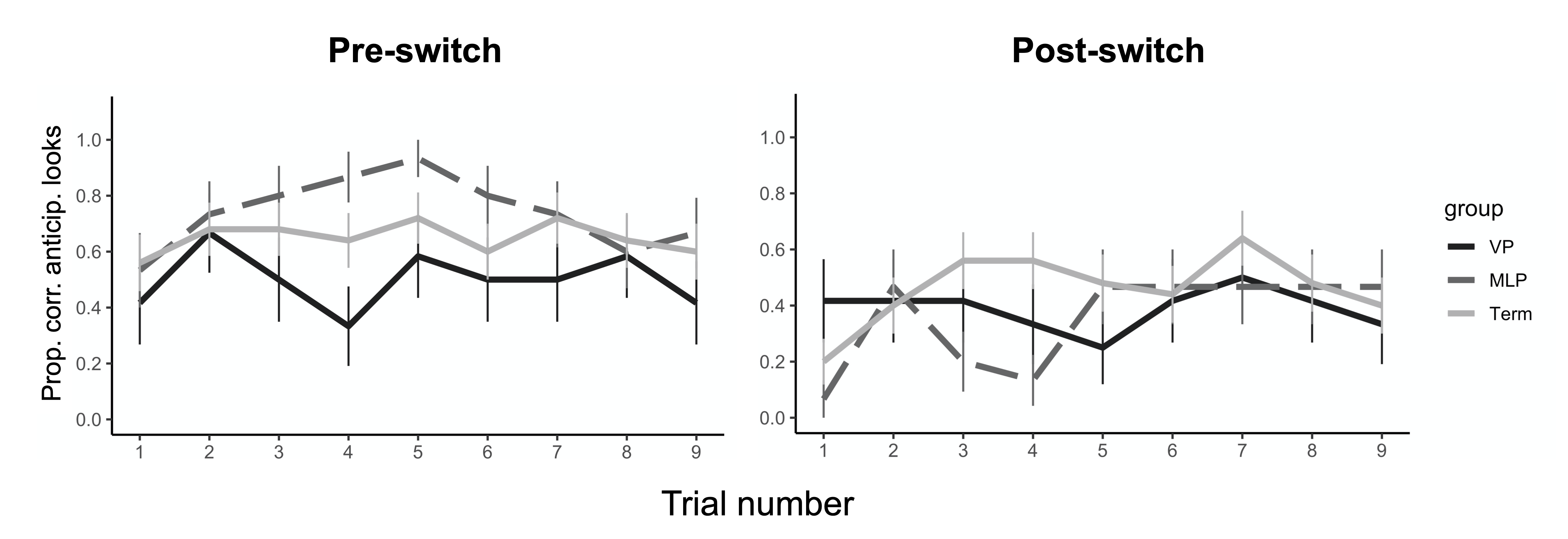


***Figure S1***. Change of change of proportion of correct anticipatory looks on the correct target in VP (*n* = 12), MLP (*n* = 15), and Term infants (*n* = 25) across 18 trials (Pre-switch phase: 1–9 trials; Post-switch phase 1–9 trials). Error bars indicate 1 standard error of the mean.

For statistical analysis, we calculated the proportion of correct anticipatory looks in each of the blocks in the pre- and post-switch phases and for each infant, respectively. We then performed a 3 (group: very preterm [VP], moderate to late preterm [MLP], term) x 2 (block; first, second) factorial analysis of variance (ANOVA) for the ratio of correct anticipatory looks as dependent variables. The factor of group was between-subjects, and the factor of block was within-subjects. In repeated-measures ANOVAs, post hoc multiple comparisons were conducted using Shaffer’s modified sequentially rejective Bonferroni procedure.

As a result, we found that there were no significant effects or interactions in either the pre- or post-phase (all *p* >.10; **Figure S2**), although the mean value of the proportion of correct anticipatory looks in the VP group was lower than MLP in the first and second blocks of pre-phase and the first half of post-phase, than in the term group in all the blocks. These results suggest that the categorical variables for anticipatory looks were not sensitive to the group differences related to neurobehavioural outcomes, in contrast to the continuous variables of looking time.

***
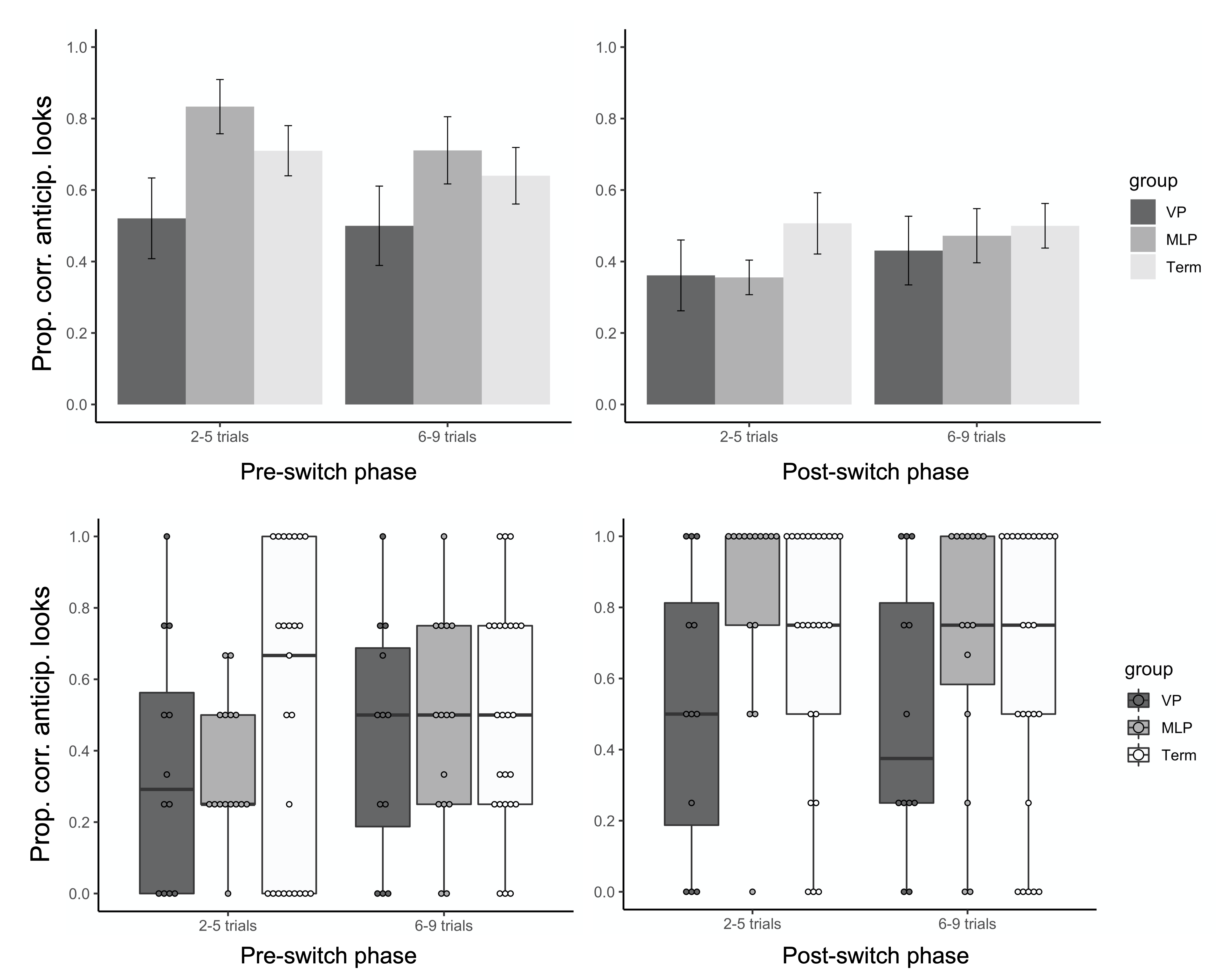
Figure S2****.* Bar (top) and box plots with scatters (bottom) indicating the proportion of correct anticipatory looks between the former (2–5 trials) and latter half (6–9 trials) of Pre/Post-switch phase in VP (*n* = 12), MLP (*n* = 15), and term infants (*n* = 25). Error bars indicate 1 standard error of the mean.

**Supplement S2: Relationships of birth profile to oculomotor response shifting at 12 months and neurobehavioural outcomes at 18 months**

We investigated the role of birth profile, including medical complications (**Table S1**), as additional factors predicting individual differences in the oculomotor response shifting at 12 months and neurobehavioural outcomes at 18 months.

We performed a correlation analysis for the continuous variable of the birth profile (i.e. gestational age and birth weight), and unpaired Student’s *t*-test for the binary variable of the birth profile (i.e. sex, small-for-gestational age [SGA][3], hyperbilirubinemia [4], hypoglycemia [5], transient tachypnea [TTN], respiratory distress syndrome [RDS] [6, 7], and periventricular echogenicities [PVE][8, 9]).

Regarding oculomotor response shifting at 12 months, we found that infants with TTN exhibited significantly lower looking time toward the correct AOI during the first block and the second block pre-switch phase (*t* = -2.21, *p* =.034; *t* = -2.20, *p* =.035) and the first block of the post-switch phase (*t* = -2.51, *p* =.016) compared to those without TTN. In addition, infants with hypoglycemia showed significantly higher looking time toward the correct AOI during the first block of the pre-switch phase (*t* = 2.76, *p* =.016). On the other hand, we did not find any other significant associations of birth profile with a decrease in looking time towards the incorrect target AOI during the post-switch phase, which was observed remarkably in the VP group. Further research is needed to investigate other factors affecting the atypical inhibition of perseverative looking in a larger sample.

Regarding the relationship with neurobehavioural outcomes at 18 months, we found that both gestational age and birth weight were significantly associated with the language-social score (L-S) in KSPD (*r* = -.34, *p* =.019; *r* = -.33, *p* =.025), and that female infants showed significantly a higher L-S score in KSPD than male infants (*t* = -2.67, *p* =.011). Regarding medical complications, infants with hyperbilirubinemia showed significantly a lower cognitive-adaptative score (C-A) in KSPD compared to those without hyperbilirubinemia (*t* = -2.26, *p* =.044). Furthermore, infants with PVE had a lower attention shifting score (AS) in the ECBQ than those without PVE (*t* = -2.78, *p* =.022). Considering the relatively high incidence of hyperbilirubinemia and PVE in preterm groups, these results suggest that the medical complications related to VP birth may be partly associated with negative neurobehavioural outcomes at 18 months [10-12].

**References**

1. Kovács, A. M. & Mehler, J. Cognitive gains in 7-month-old bilingual infants. *Proc. Natl. Acad. Sci. U. S. A.* **106**, 6556–6560 (2009)
2. Wass, S., Porayska-Pomsta, K. & Johnson, M. H. Training attentional control in infancy. *Curr. Biol.* **21**, 1543–1547 (2011)
3. Feldman, R. & Eidelman, A. I. Neonatal state organization, neuromaturation, mother-infant interaction, and cognitive development in small-for-gestational-age premature infants. *Pediatrics* 118, e869–e878 (2006)
4. Koziol, L. F., Budding, D. E. & Chidekel, D. Hyperbilirubinemia: subcortical mechanisms of cognitive and behavioral dysfunction. *Pediatr. Neurol*. 48, 3–13 (2013)
5. Goode, R. H. et al. Developmental outcomes of preterm infants with neonatal hypoglycemia. *Pediatrics* 138, (2016)
6. Lauterbach, M. D., Raz, S. & Sander, C. J. Neonatal hypoxic risk in preterm birth infants: the influence of sex and severity of respiratory distress on cognitive recovery. *Neuropsychology* 15, 411–420 (2001)
7. Patrianakos-Hoobler, A. I., Msall, M. E., Marks, J. D., Huo, D. & Schreiber, M. D. Risk factors affecting school readiness in premature infants with respiratory distress syndrome. *Pediatrics* 124, 258–267 (2009)
8. Butcher, P. R., Kalverboer, A. F., Geuze, R. H. & Stremmelaar, E. F. A longitudinal study of the development of shifts of gaze to a peripheral stimulus in preterm infants with transient periventricular echogenicity. *J. Exp. Child Psychol.* 82, 116–140 (2002)
9. Chen, C.-C., Huang, C.-B., Chung, M.-Y., Huang, L.-T. & Yang, C.-Y. Periventricular echogenicity is related to delayed neurodevelopment of preterm infants. *Am. J. Perinatol*. 21, 483–489 (2004)
10. Aarnoudse-Moens, C. S. H., Weisglas-Kuperus, N., Duivenvoorden, H. J., van Goudoever, J. B. & Oosterlaan, J. Executive Function and IQ predict mathematical and attention problems in very preterm children. *PLoS One* **8**, e55994 (2013)
11. Twilhaar, E. S., de Kieviet, J. F., van Elburg, R. M. & Oosterlaan, J. Academic trajectories of very preterm born children at school age. *Arch. Dis. Child. Fetal Neonatal Ed.* **104**, F419–F423 (2019)
12. Aarnoudse-Moens, C. S. H., Weisglas-Kuperus, N., van Goudoever, J. B. & Oosterlaan, J. Meta-analysis of neurobehavioral outcomes in very preterm and/or very low birth weight children. *Pediatrics* **124**, 717–728 (2009)
